# Diagnostic performance of corona virus disease 2019 chest computer tomography image recognition based on deep learning: Systematic review and meta-analysis

**DOI:** 10.1097/MD.0000000000031346

**Published:** 2022-10-21

**Authors:** Qiaolan Wang, Jingxuan Ma, Luoning Zhang, Linshen Xie

**Affiliations:** a West China School of Public Health and West China Fourth Hospital, Sichuan University, Chengdu, China; b West China-PUMC C.C. Chen Institute of Health, Sichuan University, Chengdu, China.

**Keywords:** chest CT, corona virus disease 2019, deep learning

## Abstract

To analyze the diagnosis performance of deep learning model used in corona virus disease 2019 (COVID-19) computer tomography(CT) chest scans. The included sample contains healthy people, confirmed COVID-19 patients and unconfirmed suspected patients with corresponding symptoms.

**Methods:**

PubMed, Web of Science, Wiley, China National Knowledge Infrastructure, WAN FANG DATA, and Cochrane Library were searched for articles. Three researchers independently screened the literature, extracted the data. Any differences will be resolved by consulting the third author to ensure that a highly reliable and useful research paper is produced. Data were extracted from the final articles, including: authors, country of study, study type, sample size, participant demographics, type and name of AI software, results (accuracy, sensitivity, specificity, ROC, and predictive values), other outcome(s) if applicable.

**Results:**

Among the 3891 searched results, 32 articles describing 51,392 confirmed patients and 7686 non-infected individuals met the inclusion criteria. The pooled sensitivity, the pooled specificity, positive likelihood ratio, negative likelihood ratio and the pooled diagnostic odds ratio (OR) is 0.87(95%CI [confidence interval]: 0.85, 0.89), 0.85(95%CI: 0.82, 0.87), 6.7(95%CI: 5.7, 7.8), 0.14(95%CI: 0.12, 0.16), and 49(95%CI: 38, 65). Further, the AUROC (area under the receiver operating characteristic curve) is 0.94(95%CI: 0.91, 0.96). Secondary outcomes are specific sensitivity and specificity within subgroups defined by different models. Resnet has the best diagnostic performance, which has the highest sensitivity (0.91[95%CI: 0.87, 0.94]), specificity (0.90[95%CI: 0.86, 0.93]) and AUROC (0.96[95%CI: 0.94, 0.97]), according to the AUROC, we can get the rank Resnet > Densenet > VGG > Mobilenet > Inception > Effficient > Alexnet.

**Conclusions:**

Our study findings show that deep learning models have immense potential in accurately stratifying COVID-19 patients and in correctly differentiating them from patients with other types of pneumonia and normal patients. Implementation of deep learning-based tools can assist radiologists in correctly and quickly detecting COVID-19 and, consequently, in combating the COVID-19 pandemic.

## 1. Introduction

Nowadays, the corona virus disease 2019 (COVID-19) has become the most urgent public health issue in the world. The COVID-19 outbreak continues to constitute a “public health emergency of international concern,” according to a statement released by World Health Organization at the seventh COVID-19 Emergency Committee meeting. Globally, as of September 11, 2022, over 605 million confirmed cases and over 6.4 million deaths, reported to World Health Organization. The number of new COVID-19 deaths worldwide has also increased for 5 consecutive weeks, bringing the cumulative number of deaths to more than 3 million. The number of people aged 25 to 59 diagnosed and admitted to hospital is “increasing at an alarming rate,” possibly due to the spread of the mutant novel corona virus strain and the social clustering of young people. A large number of patients carried unexpected economical burdens and caused health problems, moreover, the rapid speed of transmission brought panic and instability to the whole world. However, there is no recognized specific drug to cure the disease. The medical staff are facing more and more challenges such as the rapid diagnosis of patients, reasonable treatment of patients, and patient prognosis management.

Nucleic acid testing is crucial for the diagnosis of COVID-19. Some scholars pointed out that the nucleic acid testing is not the only means of diagnosis, for nucleic acid testing negative and has direct or indirect exposure history and clinical behavior, should immediately take a chest CT examination, if showing typical, it can be classified as confirmed cases, if not, the suspected infected person should also be in quarantine. Some scholars recommended chest CT imaging as the main diagnostic basis for 2019-nCoV pneumonia. With the rapid development of high and new technologies in China, the field of artificial intelligence is also promoted. Image recognition is an important subject in the field of artificial intelligence, which mainly includes 2 modules: classification recognition and feature extraction. Meanwhile, as an important research direction of artificial intelligence, deep learning has made great progress in recent years. It is widely used in image recognition and has achieved great success. Some studies have already applied deep learning to the recognition of chest CT imaging in order to obtain better diagnostic results. In this study, we aim to discuss the diagnosis performance of deep learning model used in COVID-19 CT chest scans.

## 2. Methods

This meta-analysis was carried out in accordance with the Preferred Reporting Items of Systematic Reviews and Meta-Analyses (PRISMA) guidelines. This systematic review was registered with PROSPERO, registration number CRRD 420221433. The review protocol can be find on PROSPERO (https://www.crd.york.ac.uk/prospero/), any interpretation and modification of this protocol can be viewed on this website, which has been disseminated. All analyses were based on previous published studies, thus no ethical approval and patient consent are required.

The primary procedures were as follows.

### 2.1. Criteria for considering studies for this review

Inclusion criteria: Similar research hypotheses and methods; The number of years of research development or publication; The sample size was clearly stipulated in all studies; There are clear criteria for the selection of patients, diagnosis and staging of cases in each study. Sample size, true positive number, false positive number, false negative number and true negative number can be provided. Exclusion criteria: Repeated reports; There are defects in research design and poor quality; Incomplete data and unclear outcome effect; The statistical method was wrong and could not be corrected, and cannot be provided or converted into sensitivity, specificity and accuracy.

### 2.2. Search methods for identification of studies

Three authors will independently search the relevant literature in the following electronic databases: PubMed, Web of Science, Wiley, China National Knowledge Infrastructure, WAN FANG DATA, and Cochrane Library. The selection of studies was developed with the systematic review management platform COVIDENCE. The identified studies were first stored and checked for duplicates. This review was carried out following the “Preferred Reporting Items for Systematic reviews and Meta-Analyses, PRISMA.”

The search was conducted for publications in the English language. The retrieval was a combination of subject words and free words, and the coded keywords were as follows: (“deep Learning” AND “CT” AND “Covid-19”) AND (“machine learning” AND “CT” AND “Covid-19”) AND (“machine learning” AND “diagnose” AND “Covid-19”) AND (“machine learning” AND “diagnose” AND “Covid-19”).

### 2.3. Data collection and analysis

We reviewed the titles, abstracts, and full texts of manuscripts by duplicate removal based on the above-mentioned selection criteria. The abstracts of identified articles were separately reviewed by 2 readers. After we confirmed the inclusion of associated documents, we independently extracted following variables, including the name of the first author, publication year, number of patients, and study area. All included literature was evaluated using the Quality Assessment of Diagnostic Accuracy Studies Tool. Data extraction and quality assessment were carried out independently by 2 reviewers. In case of disagreement, consensus was reached by discussing with a third reviewer.

For all clinical outcomes, individual patients were considered as the unit of analysis. For diagnostic accuracy, the sensitivity and specificity were calculated as summary measures. All the statistical analyses were carried out using Stata statistical software version 15.0. The proportions of various CT features in each group were analyzed as follows: original data were transformed by double arcsine method in Stata at first and the final conclusions were drawn using restoring formula (*P* = (sin(tp/2))2). The association between the CT features and the severity of COVID-19 pneumonia was assessed in the form of odds ratio (OR) at a 95% confidence interval (95%CI). Heterogeneity among each study was evaluated using Cochran’s *Q* test and Inconsistency index (*I*^2^) test (Table 1). *I*^2^ > 50% indicates the apparent heterogeneity between the studies and the random effects model (Der Simonian and Laird method) would be adopted. We visually assessed between-study heterogeneity by plotting the accuracy estimates in the receiver operating characteristic curve space.

**Table 1:** Results of the test of heterogeneity.

Model	Studies	Reference-positive units	Reference-negative units	Correlation	Proportion of heterogeneity likely due to threshold effect
Alexnet	5	885	302	1	1
Densenet	17	15,831	2087	0.48	0.23
Efficientnet	8	1001	265	0.71	0.5
Inception	11	2494	522	−0.87	0.76
Mobilenet	7	2735	399	−1	1
Resnet	36	14,356	1931	0.63	0.39
VGG	15	3047	485	0.36	0.13

This table is shown to present the models’ information of the heterogeneity. After the Cochran’s *Q* test and inconsistency index (*I*^2^) test, the statistics are shown in the table. The number of studies, the number of reference-positive units, the number of reference-negative units, the correlation and proportion of heterogeneity likely due to threshold effect of each model is explained.

Otherwise, the mixed model would be used. Publication bias was assessed for CT characteristics that included more than 10 studies using funnel plots and Harbord’s tests. Deviation from the funnel-shaped distribution of eligible research works suggested the presence of publication bias.

In this study, the Quality Assessment of Diagnostic Accuracy Studies-2 tool was used for migration risk assessment, which consisted of 4 parts: the selection of cases; the trial to be evaluated; the gold standard; and the process and progress of cases. In this study, strict gold standard tests were used as the basic conditions for literature screening, so there was basically no risk of bias on the part of the gold standard.

## 3. Results

**Figure 1.**
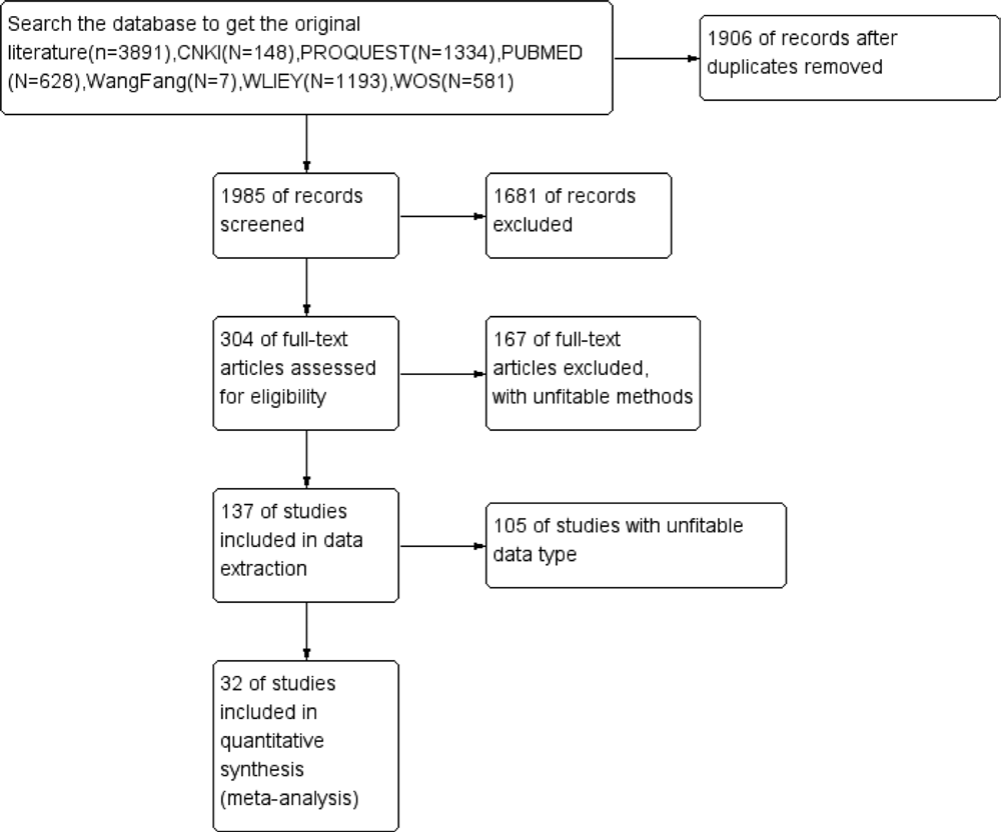
Summary of article selection process.

**Figure 2.**
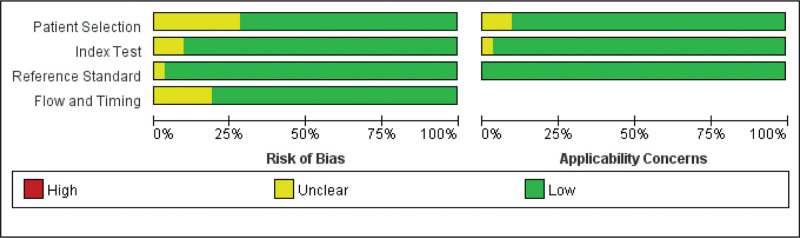
Methodological quality of included studies.

**Figure 3.**
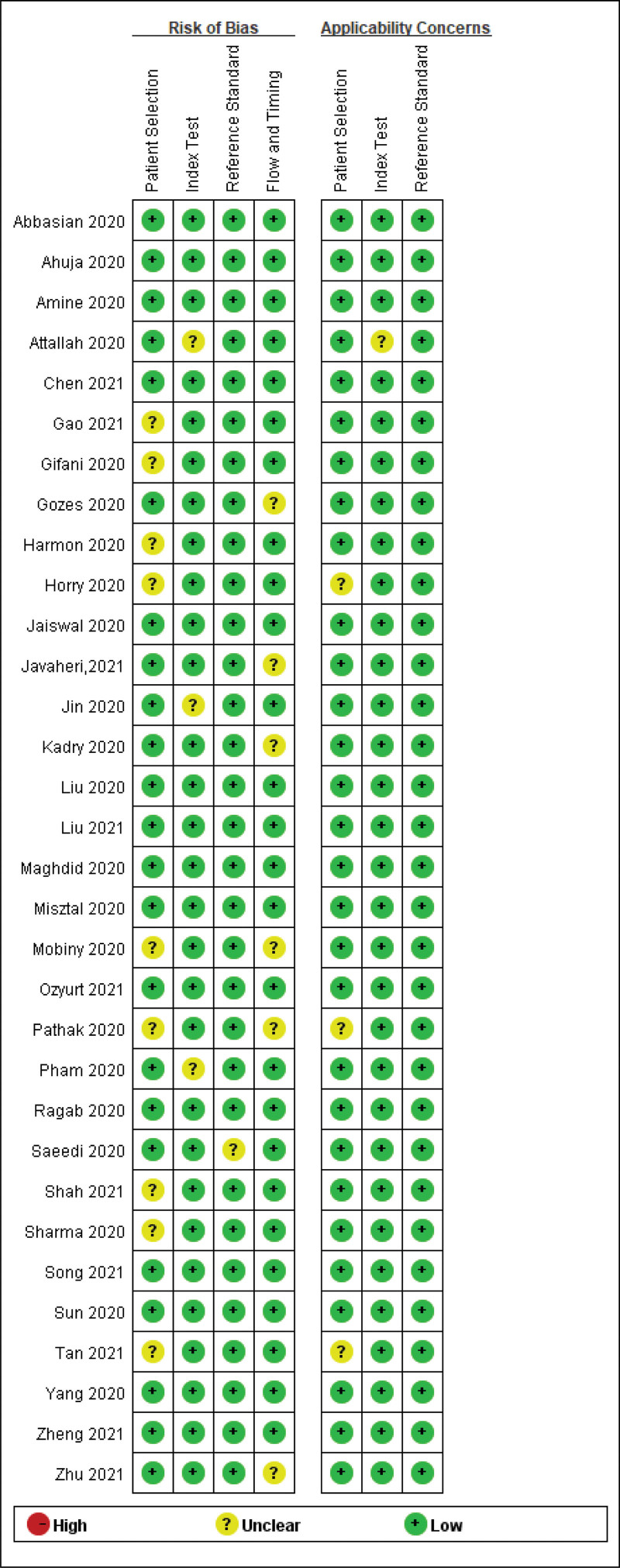
Methodological quality summary.

From the databases mentioned above, we retrieved 3891 articles. After removing 1906 duplicated articles, 1985 articles remained. After reading the titles and abstracts, 1681 papers were excluded. After reading the full text, we kept 32 descriptive studies including 51392 COVID-19 pneumonia patients in this meta-analysis.^[1–32]^ The entire process was shown in Figure 1. All the included studies were retrospective studies. The primary characteristics of the literature were exhibited in Tables 2 and 3. Generally speaking, these articles were considered to be of good quality. The result of the evidence grade was presented in the fellow figures (Figs. 2 and 3).

**Table 2:** The characteristics of the literatures.

Model	Research
AlexNet	Amine, 2020; Attallah, 2020; Maghdid, 2020; Pham, 2020; Ragab, 2020
ResNet	Sakshi, 2020; Amine, 2020; Attallah, 2020; Gozes, 2020; Jaiswal, 2020; Gifani, 2020; Jin, 2020; Misztal, 2020; Mobiny, 2020; Pathak, 2020; Pham, 2020; Ragab, 2020; Sharma, 2020; Saeedi, 2020; Chen, 2021; Gao, 2021; Shah, 2021; Song, 2021; Zheng, 2021; Zhu, 2021
DenseNet	Amine, 2020; Harmon, 2020; Jaiswal, 2020; Gifani, 2020; Misztal, 2020; Mobiny, 2020; Pham, 2020; Sharma, 2020; Saeedi, 2020; Yang, 2020; Liu, 2021; Shah, 2021; Song, 2021; Zheng, 2021
EfficientNet	Amine, 2020; Gifani, 2020
Inception	Amine, 2020; Jaiswal, 2020; Gifani, 2020; Mobiny, 2020; Pham, 2020; Sharma, 2020; Saeedi, 2020; Shah, 2021
MobileNet	Pham, 2020; Sharma, 2020; Saeedi, 2020
VGG	Amine, 2020; Jaiswal, 2020; Horry, 2020; Pham, 2020; Shah, 2021; Song, 2021; Tan, 2021; Zheng, 2021; Zhu, 2021
GoogleNet	Attallah, 2020; Pham, 2020; Ragab, 2020; Zhu, 2021
DT(decision tree)	Ali,2020; 2020, Kadry; 2020, Liu
RF(random forests)	Sun, 2020
NASNet	Gifani, 2020; Pham, 2020
ShuttleNet	Attallah, 2020; Pham, 2020; Ragab, 2020
ANN	Pathak, 2020; Ozyurt,2021
SqueezeNet	Pham, 2020
BCDU-Net	Javaheri, 2021

This table aims to present the use of algorithms in the included papers.

**Table 3:** The characteristics of the patients included in this study.

ID	Study (author, yr)	Country	Sample	Patient	Normal people	Model	Inputs	Outputs
1	Abbasian, 2020	Iran	612	306	306	K-nearest neighbor, DTL, ensemble model,	Chest HRCT	Classification (COVID-19; non-COVID-19)
2	Ahuja, 2020	India	406	95	72	ResNet, SqueezeNet	CT scans	Classification (COVID-19; non-COVID)
3	Amyar, 2020	France	150	50	100	CNN,DenseNet, ensemble model, ResNet, AlexNET, VGG, EffcientNet, Inception V3	CT scans	Classification (Covid-19+; Normal; Others) + Two images (Image reconstruction; Infection and segmentation)
4	Attallah, 2020	China	744	347	397	GoogleNet, ShuffleNet, ensemble model, AlexNET, ResNet	CT scans	Classification (COVID-19; non-COVID-19)
5	Gozes, 2020	China, US	206	56	100	ResNet	Full thoracic CT	A lung abnormality localization map; Quantitative opacity measurements
6	Harmon, 2020	China, Japan, Italy, US	2617	326	1011	DenseNet, ensemble model	Whole lung regions of CT scans	Classification (yes COVID-19; no COVID-19)
7	Jaiswal, 2020	India	374	190	184	VGG, DenseNet, ensemble model	CT scans	Classification (COVID-19 (+); COVID-19 (−))
8	Gifani, 2020	China	387	216	171	Xception, DenseNet, Inception V3, ensemble model, ResNet, EffcientNet	CT scans	Classification
9	Horry, 2020	Australia,US, China	150	81	69	VGG	X-Ray, Ultrasound, CT scan	Classification (COVID-19; Normal; Pneumonia)
10	Jin, 2020	China	2688	751	1937*	ResNet	Multichannel image, lung-masked slices	Classification (non-pneumonia; CAP; Influenza; COVID-19)
11	Kadry, 2020	Lebanon, India	500	250	250	ensemble model, DTL, Random Forst, K-nearest neighbor	CT scans	Classification (Normal; COVID-19)
12	Krzysztof, 2020	Poland	203	98	105	ensemble model, ResNet, DenseNet	Full CT lung scans, radiograph images (Front views & lateral views)	Classification (fungal pneumonia; COVID-19; healthy chest; viral pneumonia; bacterial pneumonia)
13	Liu, 2020	China	88	61	27	DTL, ensemble model, Logistic regression, K-nearest neighbor,	CT scans	Classification (COVID-19; GP)
ID	Study (author, yr)	Country	Sample	Patient	Normal people	Model	Inputs	Outputs
14	Maghdid, 2020	Iraq, UK	23	17	6	AlexNET, CNN	X-ray, CT scans	Classification (Negative; Positive)
15	Mobiny, 2020	China	105	47	58	Inception V3, DenseNet, ResNet	X-ray, CT scans	Classification (Negative; Positive)
16	Pathak, 2020	India	530	270	260	CNN, DTL, ResNet	Chest CT images	Classification
17	Pham, 2020	US	746	349	397	Inception V3, ensemble model, AlexNET,VGG,ResNet,MoblieNet,ShuffleNet,DenseNet,GoogleNet, SqueezeNet,Xception	Chest CT images	Classification (COVID+COVID-)
18	Ragab, 2020	Brazil	120	60	60	ensemble model, AlexNET, ResNet, GoogleNet, ShuffleNet	Whole CT image slices	Classification (COVID-19 pneumonia; Healthy)
19	Sharma, 2020	Italy,India,China, Moscow	2200	1400†	800	Inception V3 ensemble model, DenseNet, MoblieNet	CT scans	Classification (COVID-19; non-COVID-19)
20	Saeedi, 2020	China	746	349	397	Inception V3, ensemble model, DenseNet, MoblieNet	CT scans (COVID-19 CT scans showing typical patches on the outer edges of the lung)	Classification (COVID-19; Normal health; Other viral pneumonia)
21	Yang, 2020	China	295	70	70	DenseNet	CT scans	Classification (COVID; Non-COVID)
22	Zheng, 2021	China	659	262	397‡	DenseNet, ResNet, VGG	CT scans	Classification (Patients; Healthy person)
23	Chen, 2021	China	610	39	53§	ResNet	CT images (whole lung, include the chest wall and armpits on both sides)	Classification (Healthy; COVID-19; Bacterial Pneumonia; Typical Viral Pneumonia) (Image-level and human-level)
24	Gao, 2021	China	1202	656	423	ResNet, CNN, VGG	CT scans	Classification (COVID-19; Normal control; Other pneumonias)
25	Javaheri, 2021	US,Iran, Canada	335	226∥	109	CNN	Thick-section CT scans	Classification (Covid-19; normal) (image level and individual level) segmentation of lesions; FCN
26	Liu, 2021	China	2800	233	289	DenseNet	3D CT images	Classification (Covid-19; CAP; Control)
ID	Study (author, yr)	Country	Sample	Patient	Normal people	Model	Inputs	Outputs
27	Ozyurt, 2021	China	746	349	397	CNN, DNN	A stack of 64 axial images of size 384 of whole chest CTs	Classification (COVID-19 pneumonia; non-COVID-19 pneumonia)
28	Shah, 2021	US	73	34	39	Inception V3, VGG, ensemble model, DenseNet, ResNet	Chest CT images	Classification (COVID-19; Healthy)
29	Song, 2021	China	274	188¶	86	VGG, ensemble model, DenseNet, ResNet	CT scans	Classification (COVID-19 positive; COVID-19 negative)
30	Tan, 2021	China	470	275	195	VGG	Chest CT images	Classification (COVID-19; Bacteria pneumonia) (image-level prediction and individual-level prediction)
31	Zhu, 2021	China	1592	275	235	VGG, ResNet, GoogleNet	CT scans	Classification (COVID-19; Normal)

The table is shown to the summaries of the characteristics of the patients included in this study including demographics, clinical features and the inputs and outputs of the models.

COVID-19 = corona virus disease 2019.

*including 1229 non-pneumonia, 668 CAP, 42 Influenza.

†including 800 COVID-19, 600 Other viral pneumonia.

‡including 100 bacterial pneumonia, 219 typical viral pneumonia, 78 healthy.

§including 38 other pneumonias, 15 normal controls.

∥including 111 infections with CAP and 115 other viral sources, whose CT images may be misdiagnosed as COVID-19.

¶including 88 COVID-19, 100 patients infected with bacteria pneumonia.

**Figure 4.**
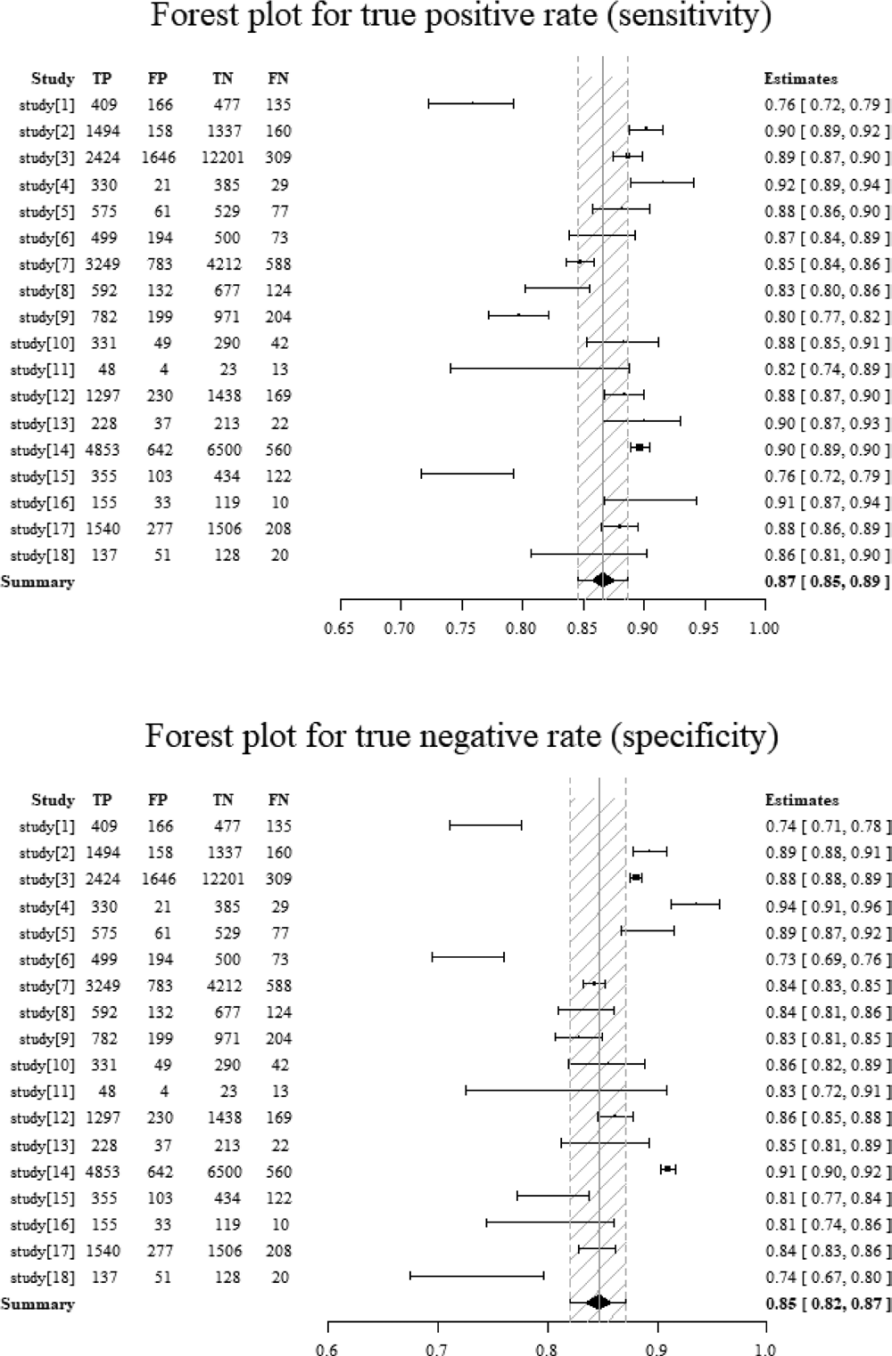
Forest plot of the deep learning Diagnostic method.

**Figure 5.**
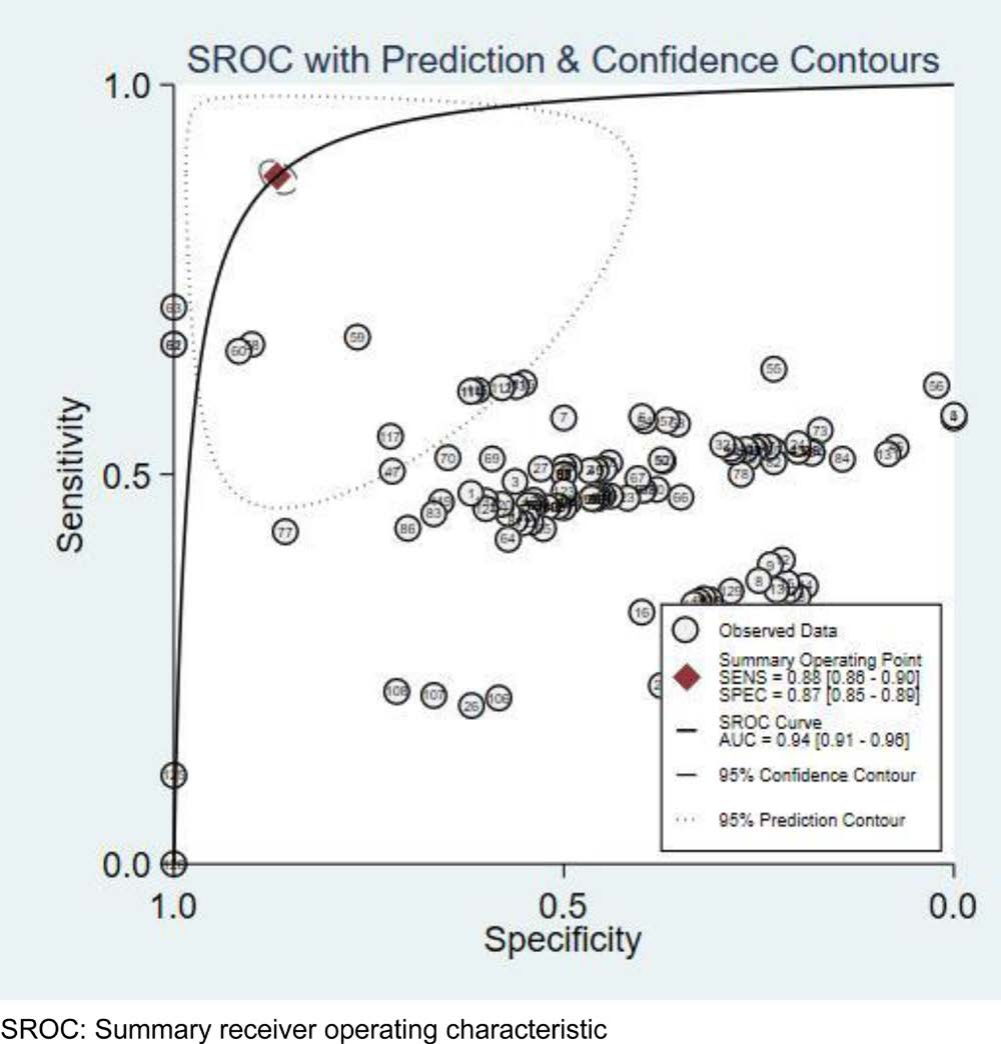
Summary receiver operating characteristic (SROC) curve of the deep learning method.

**Figure 6.**
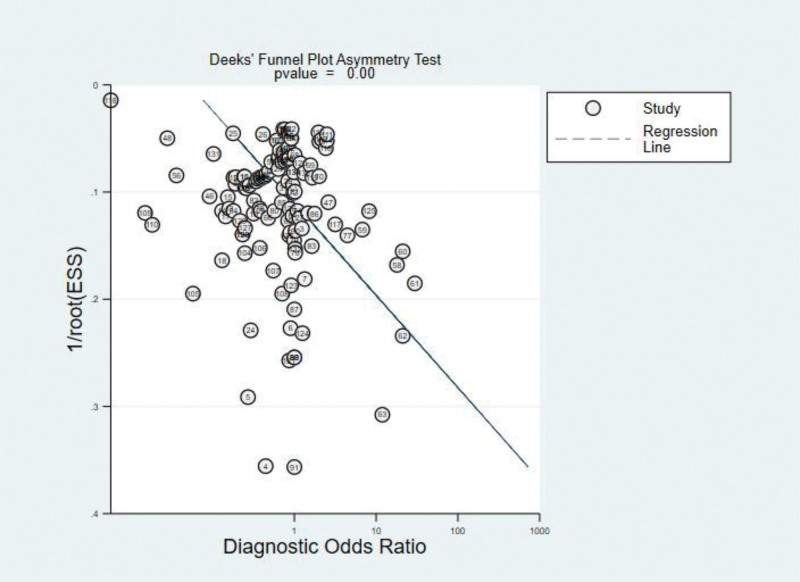
Deek’s funnel plot asymmetry test.

After analyzed data from the selected literature, the diagnostic performance of deep learning models was measured by the combined sensitivity (0.87[95%CI: 0.85, 0.89]), combined specificity (0.85[95%CI: 0.82, 0.87]) (Fig. 4), combined positive likelihood ratio (6.7[95%CI: 5.7, 7.8]), combined negative likelihood ratio (0.14[95%CI: 0.12, 0.16]) and diagnostic OR (49[95%CI: 38, 65]), the area under the receiver operating characteristic curve (AUROC) was 0.94(95%CI: 0.91, 0.96) (Figs. 5 and 6). However, after the statistical test for publication bias, the *T* value was 6.68 (*P* < .05), and there remained publication bias in the included literature. The result of the test of heterogeneity showed the Q value is 26815.83 (*P* < .05), and the *I^2^* score is 99.5%, which indicated the high heterogeneity. So the analysis of the subgroup was necessary, we could calculate the combined sensitivity, combined specificity, combined positive likelihood ratio, combined negative likelihood ratio, combined diagnostic OR and AUROC of each model. According to the table, Resnet has the best performance, which has the highest sensitivity (0.91[95%CI: 0.87, 0.94]), specificity (0.90[95%CI: 0.86, 0.93]), positive likelihood ratio (8.9[95%CI: 6.2, 12.9]), diagnostic OR (89[95%CI: 43, 187]) and AUROC (0.96[95%CI: 0.94, 0.97]), then Densenet was seems to be the second best choice for the diagnosis, although having the same AUROC (0.93[95%CI: 0.91, 0.95]) compared with VGG, the former has a higher specificity (0.87[95%CI: 0.80, 0.92]) and diagnostic OR (45[95%CI: 22, 95]).Considering the same AUROC (0.9[95%CI: 0.88, 0.93]), Mobilenet has higher sensitivity (0.89[95%CI: 0.87, 0.9]), specificity (0.86[95%CI: 0.82, 0.89]) and diagnostic OR (47[95%CI: 34, 64]) than Inception and Efficientnet. Alexnet, whose AUROC was 0.86(95%CI: 0.83, 0.89), has lower sensitivity (0.79[95%CI: 0.66, 0.88]), specificity (0.80[95%CI: 0.65, 0.90]) and diagnostic OR (15[95%CI: 4, 61]) (Table 4).

**Table 4:** Diagnostic performance of the including models.

Model	Combined sensitivity (95%CI)	Combined specificity (95%CI)	Combined positive LR (95%CI)	Combine NegativeLR (95%CI)	Combined DOR (95%CI)	AUROC (95%CI)
Alexnet	0.79(0.66, 0.88)	0.80(0.65, 0.90)	4(1.9, 8.5)	0.26(0.14, 0.50)	15(4, 61)	0.86(0.83–0.89)
Densenet	0.87(0.83, 0.91)	0.87(0.80, 0.92)	6.6(4.1, 10.7)	0.15(0.11, 0.21)	45(22, 95)	0.93(0.91, 0.95)
Efficientnet	0.88(0.84, 0.9)	0.73(0.66, 0.78)	3.2(2.5, 4)	0.17(0.13, 0.23)	19(12, 30)	0.9(0.87, 0.92)
Inception	0.81(0.61 ,0.92)	0.86(0.78, 0.92)	5.8(4.0, 8.4)	0.23(0.11, 0.47)	26(14, 49)	0.9(0.88, 0.93)
Mobilenet	0.89(0.87, 0.9)	0.86(0.82, 0.89)	6.3(4.9, 8.0)	0.13(0.12, 0.16)	47(34, 64)	0.9(0.87, 0.93)
Resnet	0.91(0.87, 0.94)	0.9(0.86, 0.93)	8.9(6.2, 12.9)	0.1(0.06, 0.16)	89(43, 187)	0.96(0.94, 0.97)
VGG	0.87(0.82, 0.92)	0.86(0.79, 0.91)	6.4(4.1, 10)	0.15(0.1, 0.22)	44(20, 94)	0.93(0.91, 0.95)
TOTAL	0.87(0.85, 0.89)	0.85(0.82, 0.87)	6.67(5.74, 7.76)	0.14(0.12, 0.16)	49(38, 65)	0.94(0.91, 0.96)

This table is shown to present the models’ information of the diagnostic performance. After the statistical analysis, the statistics are shown in the table. The combined sensitivity, specificity, positive likelihood ratio, negative likelihood ratio, diagnostic odds ratio and AUROC of each model is explained.

AUROC = area under the receiver operating characteristic curve, CI = confidence interval; DOR = diagnostic odds ratio; positive LR = positive likelihood ratio; negative LR = negative likelihood ratio.

## 4. Discussion

There has been much discussion about the diagnosis of COVID-19. Currently, the methods used to diagnose the pneumonia include real-time reverse transcription-polymerase Chain Reaction (RT-PCR), isothermal nucleic acid amplification assays, rapid diagnostics tests, enzyme-linked immunosorbent assay, chemiluminescent immunoassay, chest X-ray, chest CT, etc.^[33]^ At the beginning of the outbreak, some researchers have debated the accuracy and utility of forementioned methods. According to a report of 1014 COVID-19 cases, Tao found the sensitivity of chest CT in suggesting COVID-19 was 97% based on positive RT-PCR results.^[34]^ Furthermore, there is a meta-analysis showing the overall diagnostic sensitivity of 87% for chest CT. The sensitivity of RT-PCR in detecting COVID-19 was reported lower than that of chest CT. Chest CT scans can also present the progression of the disease.^[35]^ Therefore, compared with RT-PCR, chest CT scan may have beneficial diagnostic characteristics as an auxiliary diagnostic tool.^[36]^ We can draw a conclusion that chest CT may be the main tool for COVID-19 detection in the current epidemic area.

RT-PCR acquires the merits such as: high specificity – when the target gene is correctly chosen, the specificity can nearly reach 100%.^[37]^ Several different samples can be used for nucleic acid testing using RT-PCR, Nasopharyngeal and/or oropharyngeal swabs are usually recommended for screening or diagnosing early infection.^[38]^ Also, other biological samples like urine^[39]^ and feces^[40]^ are acknowledged by more and more medical staff. RT-PCR is still regarded as the golden diagnostic standard for COVID-19. However, RT-PCR has its own defects, misdiagnoses occur sometimes. First, at the early stage, a true patient can be tested negative by only nucleic acid testing.^[41,42]^ Besides, RT-PCR has high requirements for the detective equipment and platform, laboratory environment and the testing staff’s operation. Thirdly, considering the variations in the transportation and backlog, nucleic acid testing takes a long time, typically the shortest time to report the results is usually 24 hours.

Compared with other methods, CT also has particular advantages and drawbacks. As many clinicians prompting, chest CT scans not only can give a quantitative result but also present the development of the pneumonia efficiently. The specific imagining features such as ground-glass opacities and consolidation can^[43]^ be helpful in the process of recognizing the patients. In addition to time-saving, low cost and non-invasion, chest CT scans can be transmitted digitally, and doctors nationwide who specialize in radiology can be mobilized to make joint judgments. However, the diagnostic accuracy of chest CT scan results is influenced by a physician’s workload and expertise. Sometimes, patients with asymptomatic infection or early lung disease cannot be diagnosed by chest CT examination. CT examination should be used when there are typical respiratory symptoms, especially dyspnea, and murmurs in the lungs during clinical examination, then the detection rate of CT examination can be relatively higher. So, when there is the need to distinguish the suspected patients and real infected patients, chest CT examination may be a wise choice.^[44]^

The combination of medicine and other technologies is more and more popular. Many problems can be solved because of the collaboration conducted by the specialists from different areas. Deep learning is a new research direction in the field of Machine Learning. It is introduced into Machine Learning to make it closer to the original goal – artificial intelligence. In recent years, deep learning, especially convolutional neural networks, has rapidly developed into a hotspot of medical image analysis. Facing the grim situation of COVID-19, there are emerging studies designed to exploit deep learning in the process of diagnosis. Deep learning algorithm contains supervised, semi-supervised, and unsupervised learning. In the diagnosis process of COVID-19, supervised learning is the most popular. Based on supervised learning, an optimal model is obtained through training of existing training samples, and then all inputs are mapped into corresponding outputs by this model, and simple judgments are made on the outputs to achieve the purpose of prediction and classification, thus having the ability to predict and classify unknown data. The patients who have already been diagnosed infected or not can provide their chest CT scans as the training set required by the model. In order to find out the model with the best effect, a valid set is proposed to adjust the model parameters. And we can measure the performance and classification capability of the optimal model by the test set.

The deep learning models such as Resnet, Densenet, Mobilenet, VGG, Inception have been applied to diagnose lung diagnosis. During the outbreak of COVID-19, some models can achieve an accuracy nearly 100% in classifying COVID-19 positive cases from combined Pneumonia and healthy cases.^[45–47]^ According to the review, Resnet has the best performance in detecting the nidus, however, this finding is also confirmed in previous studies.^[48]^ Some studies indicated the combination of different models can improve the speed and efficiency.^[49]^ However, due to the complexity of the combined model, researchers will make some adjustments, which will lead to the poor effect of the constructed model when extrapolated. We believe that when the number of combination models is large enough, the diagnostic efficacy of combination models can be learned.

Here might be the strengths of our study: Firstly, the review was completed strictly according to the Cochrane PRISMA. Besides, we compared each model’s diagnostic indicators, so we could determine the most appropriate model which would be helpful for policy makers in considering an automated classification system in real-world clinical settings in order to speed up routine examination. However, there still remains some weakness in this study. First of all, the heterogeneity could not be ignored, the confirmed patients contained different countries, the non-COVID19 samples may have different compositions, such like all healthy people or suspected individuals (people with community-acquired pneumonia, lung cancer, tuberculosis etc). Also, the imaging levels of the CT device could be the impact factor to make the heterogeneity significant. Secondly, according to each model, the included literature was still not enough to draw an absolutely correct conclusion, we could go on continue collecting relative articles to have a more convincing result. Thirdly, publication bias existed because some small studies and negative results were not easy to publish, and to some extent, our retrieval strategy has not reached a certain efficiency. There should be follow-up work to refine the review, including developing a better search strategy, expanding the number of included articles, etc. Furthermore, we did not evaluate the ensemble models many studies proposed. The ensemble models have different types and some studies didn’t explain them explicitly, so we had to keep a cautious sight. With more relative studies’ publications, the performance of the ensemble model will be evaluated correctly.

## 5. Conclusion

In this study, we evaluated the performance of the deep learning model regarding detection of COVID-19 automatically using chest images to assist with proper diagnosis and prognosis. The findings of our study showed that the deep learning model achieved high sensitivity and specificity (88% and 87%, respectively) when detecting COVID-19. The pooled summary receiver operating characteristic curve value of both COVID-19 and other types of pneumonia was 94%. Our study findings showed that deep learning models have immense potential in accurately stratifying COVID-19 patients and in correctly differentiating them from patients with other types of pneumonia and normal patients. Implementation of deep learning-based tools can assist radiologists in correctly and quickly detecting COVID-19 and, consequently, in combating the COVID-19 pandemic.

## Author contributions

Qiaolan Wang designed the meta-analysis, extracted data and interpreted literatures. Jingxuan Ma and Luoning Zhang were responsible for the reliability of the data, checking and evaluating the quality of the collected data. Linshen Xie supervised, reviewed and revised the articles.

**Conceptualization:** Linshen Xie.

**Data curation:** Qiaolan Wang, Jingxuan Ma, Luoning Zhang.

**Formal analysis:** Qiaolan Wang.

**Funding acquisition:** Jingxuan Ma.

**Investigation:** Jingxuan Ma, Luoning Zhang.

**Methodology:** Qiaolan Wang, Jingxuan Ma.

**Project administration:** Qiaolan Wang.

**Software:** Qiaolan Wang.

**Supervision:** Linshen Xie.

**Writing – original draft:** Qiaolan Wang.

**Writing – review & editing:** Qiaolan Wang.
